# Three Years of Salvage IMRT for Prostate Cancer: Results of the Montpellier Cancer Center

**DOI:** 10.5402/2012/391705

**Published:** 2012-03-20

**Authors:** Olivier Riou, Pascal Fenoglietto, Benoit Laliberté, Cathy Menkarios, Carmen Llacer Moscardo, Meng Huor Hay, Norbert Ailleres, Jean-Bernard Dubois, Xavier Rebillard, David Azria

**Affiliations:** ^1^Département d'Oncologie Radiothérapie, CRLC Val d'Aurelle-Paul Lamarque, Montpellier 34298, France; ^2^Département de Radio-Oncologie, Hôpital Maisonneuve-Rosemont, Montréal, QC, Canada H1T 2M4; ^3^Department of Urology, Clinique Beausoleil, Montpellier 34070, France

## Abstract

*Background*. To assess the feasibility of salvage intensity-modulated radiation Therapy (IMRT) and to examine clinical outcome. *Patients and Methods*. 57 patients were treated with salvage IMRT to the prostate bed in our center from January, 2007, to February, 2010. The mean prescription dose was 68 Gy in 34 fractions. Forty-four patients received concomitant androgen deprivation. *Results*. Doses to organs at risk were low without altering target volume coverage. Salvage IMRT was feasible without any grade 3 or 4 acute gastrointestinal or urinary toxicity. With a median follow-up of 21 months, one grade 2 urinary and 1 grade ≥2 rectal late toxicities were reported. Biological relapse-free survival was 96.5% (2.3% (1/44) relapsed with androgen suppression and 7.7% (1/13) without). *Conclusion*. Salvage IMRT is feasible and results in low acute and chronic side-effects. Longer follow-up is warranted to draw conclusions in terms of oncologic control.

## 1. Background

Any rising prostate-specific antigen (PSA) level above 0.2 ng/mL in patients who have undergone prior radical prostatectomy (RP) should be considered as a recurrence [1, 2]. Because the site of relapse, either local or distant, is sometimes equivocal [3, 4], absolute PSA value and PSA kinetic parameters such as PSA velocity at recurrence [5], interval to PSA failure [6], postoperative PSA doubling time [7–9], and preoperative PSA velocity [10] are often used to assess clinical outcome, although not always suitable to predict the type of relapse. In case of local relapse, salvage radiation therapy is able to cure selected patients [11, 12]. Nevertheless, given the risk of morbidity associated with the use of conventional three-dimensional conformal radiation therapy (3D-CRT) in the postoperative setting [13–15], most clinical trials have investigated the delivery of low-level radiation doses ranging from 60 to 66 Gy [16–18]. In contrast, more recent studies have evaluated the use of intensity-modulated radiation therapy (IMRT) in conjunction or not with image-guided RT (IGRT) at radiation doses up to 76 Gy and demonstrated acute and late toxicity profiles similar to those obtained with conventional 3D-CRT using standard doses [19].

In our institution, we decided to exploit the dosimetric advantages of IMRT, primarily not to deliver escalated doses, but to better spare the organs at risk (OAR) and to reduce the incidence of acute and late toxicities. In this paper, we present the safety results and preliminary data regarding the short-term biochemical control of patients treated with salvage IMRT to the prostatic fossa.

## 2. Methods

### 2.1. Patient Selection and Follow-Up

From January, 2007, to September 2007, the first ten patients were treated with postoperative IMRT to the prostate bed in our center. The very favorable toxicity profile observed in this series enabled us to use their dosimetric plans to set our constraints afterwards. A total of 57 consecutive patients were treated from January 2007 to February 2010 with IMRT to the prostate bed for biochemical relapse after RP. Patient characteristics are shown in Table 1. Biochemical recurrence had been established by a rise in PSA level postoperatively. Patients were seen every week during the course of treatment, and acute genitourinary (GU) and gastrointestinal (GI) toxicities were scored according to the common Toxicity Criteria for Adverse Events scale (CTCAE 3.0). After completion of the treatment, patients were followed every 3 to 6 months by routine examination and PSA test. A more frequent schedule was established in case of the occurrence of side effects. Acute toxicity was defined as any side effect that occurred during treatment or within 3 months of the initiation of radiotherapy, and the late toxicity included new or persisting symptoms occurring 3 months or later after the start of the treatment. Toxicity was reported as the highest toxicity in each patient. Biochemical relapse after salvage radiotherapy was defined as a PSA value of more than nadir +0.2 ng/mL or a continued rise in the serum PSA despite IMRT.

### 2.2. Androgen Deprivation Therapy (ADT)

The use of concomitant androgen deprivation was left to the physician's discretion, and the decision based on clinical history, pathological findings, initial staging, and patient agreement. ADT consisted of LHRH agonists, sometimes associated for the first month with anti-androgen to prevent the flare-up effect. In total, 77% of patients underwent androgen deprivation, but this treatment was short course (<9 months) in 84% of them (37/44 patients). The duration of ADT ranged from 0 to 24 months.

### 2.3. Acquisition and Simulation

Patients were instructed to empty their bladder before the scan according to our in-house protocol. They underwent computed tomography (CT)-based virtual simulation in the supine position, with knee and feet supports, but no custom immobilization device was used. The isocenter was set “online” in the middle of the prostatic fossa by the treating physician immediately after the scan, while the patient waited in the treatment position on the scan table. The isocenter was then tattooed on the patient skin.

### 2.4. Contouring and Volume Definition

Structures were manually contoured on the CT scan according to the recommendations stemming from major randomized trials, described in more detail by the EORTC Radiation Oncology Group, the Australian and New Zealand Radiation Oncology Genito-Urinary group, and the RTOG group [20–22]. The clinical target volume (CTV) included the prostate bed encompassing the vesicoureteral anastomosis, and target volume delineation was based on surgical clips, preoperative imaging, operative findings, and additional information from surgical pathology. The final length of the CTV was at least 3 cm and at most the length of the prostate gland on the pathology report or preoperative imaging. The CTV was extended to the seminal vesicles in case they were still visible on the planning CT scan and included the seminal vesicle bed in case of invasion on the RP specimen. Two planning target volumes (PTVs) were defined to account for daily set-up errors and internal motion and to provide a smooth differential dose to the CTV :  PTV1 consisted of the CTV + a 3D margin of 1 cm and PTV2 consisted of the CTV + a 3D margin of 0.5 cm. The bladder was contoured in its entirety, and the rectum as a whole organ but starting 2 cm above and below the CTV. Femoral heads were drawn from the top of the acetabulum to the small trochanter inferiorly.

### 2.5. Treatment Planning and Delivery by IMRT

Prescription doses were 68 Gy in 34 fractions for 54 patients, including the patients of the dosimetric study, and 70 Gy in 35 fractions for 3 patients. Treatment plans were generated using Eclipse software (Varian, Palo Alto, CA, USA) without heterogeneity correction. Patients were treated using either “conventional IMRT” or volumetric intensity-modulated arc therapy (RapidArc^®^). For conventional IMRT, the 3D-IMRT beam geometry consisted of five coplanar fields with gantry angles of 60°, 95°, 180°, 265°, and 300°. An 18-MV linear accelerator was used (21 EX, Varian, Palo Alto, CA, USA) and IMRT was delivered using the “sliding-window” mode of the multileaf collimator (MLC Millenium 120, Varian, Palo Alto, CA, USA). The RapidArc^®^ technique was used in the last patients (*n* = 12) with an optimization version 8.5. The treatment was delivered in a 360-degree arc with a simultaneous variation of the gantry speed, dose rate, and leaf position allowing IMRT dose distribution with reduced delivery time as compared to the fixed-gantry IMRT solution. Energy of 18 MV with a maximum dose rate of 400 monitor units per minute was used, and a collimator rotation of 45° was fixed for all patients. The minimum doses to PTV1 and PTV2 were 64 Gy and 68 Gy, respectively. IGRT was associated with IMRT using on-board digital imaging devices such as cone beam CT and kilovoltage X-ray, or megavoltage X-ray imaging.

## 3. Results

### 3.1. Dose-Volume Histograms (DVHs) and Average Dose for IMRT

**Figure 1:**
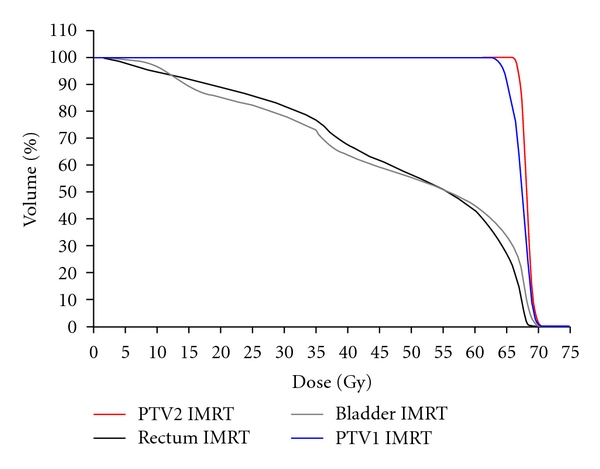
Average dose volume histogram for the first 10 patients.

The DVH generated for the first 10 patients (Figure 1) demonstrated adequate coverage of the target volumes with 95% of volume PTV1 and PTV2 receiving at least 95% of the prescribed doses (60.4 Gy and 64.6 Gy, resp.). Detailed dosimetric data are reported in Table 2 for this series. In order to obtain a cohort of homogeneously treated patients, we then applied the same constraints and reached the same dose levels for all 57 patients.

### 3.2. Acute Toxicity

As summarized in Table 3, no patient developed acute GU or GI toxicity greater than grade 2, and no patient required treatment breaks because of acute radiation-induced toxicity. Grade 2 acute toxicity was reported in five patients (8.8%) (3 GU, 1 GI, 1 both GU and GI). The most commonly observed GU symptoms were pollakiuria, nycturia, and painful urination. GI toxicity mainly included diarrhea, anal pain, and tenesmus. All reactions could be managed with symptomatic treatments in ambulatory setting.

### 3.3. Chronic Toxicity

With a median follow-up of 21 months, there was only one case of late grade 3 toxicity (Table 3). This patient developed diarrhea up to ten stools per day four months after completion of the treatment, requiring 5 days of hospitalization for rehydration and symptomatic treatment. After one month, he had recovered with regular frequency, but persisting loose stools. A single late grade 2 urinary adverse effect was observed, which was frequent urinary leakage. All other toxicities were grade 1.

### 3.4. Oncologic Control

The short-term oncologic control was very good with only 2 cases of relapse (4%) at 21 months. One patient had both biochemical and clinical relapse, with progressive nodal disease responding to androgen deprivation treatment. The other patient had biochemical failure and is currently undergoing ADT. Failure rates for patients treated with or without concomitant androgen deprivation were 2.3%  (1/44) and 7.7% (1/13), respectively.

## 4. Discussion

Postprostatectomy irradiation improves outcome either as salvage or adjuvant therapy, but at the cost of increased GI or GU toxicity compared to no irradiation. It is not clear however to what extent this treatment-related toxicity impacts on quality of life as compared to observation since the diagnosis of cancer recurrence itself, even if only biochemical, is associated with impaired physical function [23, 24]. It is also questionable whether adjuvant radiotherapy, systematically given to high-risk patients with no known recurrence, is a better cost-effective option than salvage treatment given to relapsing patients. From a clinical point of view, the superiority of one approach over the other will necessarily imply minimizing side effects [25]. The recently updated results of the EORTC 22911 trial presented at ESTRO 29 (Barcelona) and at ASTRO 2010 did not show any overall or metastasis-free survival benefit of adjuvant radiotherapy, possibly due to the use of early salvage therapy in the control arm. That is in contradiction with the results of the SWOG study [26], which had showed a reduced risk of metastasis and an increased survival in the adjuvant arm. To date, it cannot be stated whether selective salvage radiotherapy is less effective than adjuvant radiotherapy, despite a clear benefit on biochemical control in favor of adjuvant radiotherapy [17, 26–28]. Some clinical trials addressing that question are underway (RADICALS, GETUG 17) [29–31], and hopefully, the results should help us to better identify the patients who may avoid adjuvant treatment and to distinguish those who would benefit more from one treatment strategy than the other. There is clear evidence that the delivery of adjuvant radiotherapy to all patients with high-risk features will cause more radiation-related toxicity than salvage treatment only applied to those who relapse [32]. Consequently, having regard to the fact that prostatectomy for advanced stage including T3 disease is becoming common [33–35], and until the results from ongoing clinical trials are available, it can be hypothesized that salvage radiotherapy will be increasingly used, emphasizing the need for improving clinical outcome after treatment, both in terms of disease control and side-effects [36].

Unlike the large number of studies conducted for the use of dose-escalated radiation therapy as exclusive treatment of prostate cancer, this strategy has not been extensively investigated in the postoperative setting. Some randomized controlled trials have assessed adjuvant radiotherapy using conventional technique with prescription doses ranging from 60 to 64 Gy [16, 17]. In the salvage setting, doses up to 66 Gy are usually delivered with 3D-CRT, with acceptable but improvable toxicity rates. Nonetheless, it remains to be proven whether further dose escalation has beneficial effect on disease-free outcome. However, IMRT allows high doses to be delivered to the target volumes while limiting the radiation dose to the OAR [37–41]. The role of IMRT has been widely studied for the treatment of localized prostate cancer, and this treatment modality has now become a standard of care for this condition [42–46]. In contrast, there was little research in the postoperative setting because of the concerns regarding cumulative toxicity. IMRT treatment could nevertheless provide the best therapeutic ratio, since it has been shown to decrease radiation doses in normal tissues, including the rectum and bladder [47, 48]. On that basis, we introduced IMRT in our daily practice, at a dose of 68 Gy, actually not for dose escalation, but instead to minimize toxicity. Previous studies addressing the question of postprostatectomy IMRT had focused on different aspects, namely, the impact of dose escalation on clinical outcome [19, 49, 50], the effect on erectile function [51], the interest in specific radiation modalities such as the whole pelvis irradiation [52], or the comparison between adjuvant and salvage treatment [53–55]. To our knowledge, our study is the first one addressing the dosimetric feasibility and toxicity of IMRT without dose escalation for salvage treatment of prostate cancer. 

Furthermore, more than half of our patients had positive margins, which is higher than other reported studies in the salvage setting. Our results compare favorably with previously published studies, with better results than those using conventional 3D-CRT and similar findings to those using IMRT. Results are promising in terms of toxicity, but careful follow-up is required to make sure that this treatment modality would not translate into worse oncologic outcome. It is too early, and follow-up is too short to draw definite conclusions from these results with respect to disease control.

Obviously, our study has several limitations. First, the number of patient is rather low, but in return, the cohort is homogeneous in characteristics and treatment. The follow-up is adequate for acute toxicity and sufficient for late gastro-intestinal toxicity as it usually manifests within 2 years from the treatment. However, our follow-up is too early for a full assessment of urinary toxicity and much too short for disease control evaluation. Moreover, toxicity reporting using the CTCAE 3.0 scale might have underscored urgency symptoms.

Regarding the concomitant use of androgen deprivation with postoperative radiotherapy, data are conflicting, in particular for salvage IMRT. The RTOG 85-31 trial evaluated the effect of immediate androgen suppression in conjunction with standard RT versus RT alone in a group of men eligible for adjuvant treatment. This study did not allow definitive conclusions, as the results were positive for freedom from biochemical relapse, but no differences were observed for overall survival, distant failure, and local control [56]. With regard to salvage treatment, one study from the French group GETUG (GETUG 16) is addressing this question, and results are pending [57]. Initial results from the RTOG 96-01 trial presented at ASTRO meeting 2010 evaluating the benefit of adding bicalutamide to RT for salvage treatment tend to indicate an advantage for freedom from progression. On our side, we believe that patients undergoing salvage treatment should be regarded as part of a very-high-risk group, taking into consideration failure of the first treatment, potentially driving disease progression towards more aggressive forms. Until final results from ongoing trials are published, short-course concomitant androgen deprivation (<9 months) should be considered for those patients, and that is the reason why the majority of our patients currently receive this combination in this setting.

## 5. Conclusions

Salvage IMRT for rising PSA level after RP is feasible and results in very low acute and late toxicity. A longer follow-up is warranted to evaluate the impact in terms of disease control.

## Figures and Tables

**Table 1:** Patient characteristics.

Characteristics		(%)
Age, years		
Range	53 – 81	
Median	66	
PSA before RP		
Range	3.3–58.0	
Median	8.1	
≥5	46	81%
<5	11	19%
PSA after RP		
Range	0.1–1.8	
Median	0.4	
≥0.2	51	89%
<0.2	6	11%
Pathological stage		
pT2a	5	9%
pT2b	8	14%
pT2c	25	44%
pT3a	14	25%
pT3b	5	9%
pT4	0	0%
Surgical margins		
Positive	29	51%
Negative	28	49%
Gleason score		
≤6	23	40%
7	32	56%
≥8	2	4%
Type of IMRT		
5 coplanar fields	45	79%
VMAT (RapidArc)	12	21%
Androgen deprivation		
Yes	44	77%
Short course (<9 months)	37	65%
Long course	7	12%
No	13	23%

VMAT: volumetric intensity-modulated arc therapy.

**Table 2:** Dosimetric results of the first ten patients treated with salvage IMRT.

Organ or volume considered	Mean doses, Gy (range in brackets)	D50, Gy (range in brackets)
PTV1	67.2 (66.6–67.9)	
PTV2	68.1 (67.4–68.9)	
Rectum	51.4 (38.6–54.7)	54.6 (37.7–60.9)
Bladder	45.1 (39.2–62.3)	51.9 (41.2–67.4)
Femoral heads	26.1 (17.3–29.7)	

D50: dose to 50% of the organ volume.

**Table 3:** Toxicity analysis.

Side effects		Grade no. (%)		
	I	II	III	IV
Genitourinary				
Acute	19 (33)	4 (7)	—	—
Late	9 (16)	1 (2)	—	—
Gastrointestinal				
Acute	26 (46)	2 (4)	—	—
Late	4 (7)	—	1 (2)	—
